# Ancestrally Reconstructed von Willebrand Factor Reveals Evidence for Trench Warfare Coevolution between Opossums and Pit Vipers

**DOI:** 10.1093/molbev/msac140

**Published:** 2022-06-20

**Authors:** Danielle H Drabeck, Alexandra Rucavado, Erika Hingst-Zaher, Antony Dean, Sharon A Jansa

**Affiliations:** Department of Ecology, Evolution, and Behavior, University of Minnesota, 1479 Gortner Ave., St Paul, MN 55108, USA; Bell Museum, University of Minnesota, 1987 Upper Buford Circle, St. Paul, MN 55108, USA; Instituto Clodomiro Picado, Facultad de Microbiología, Universidad de Costa Rica, San José, Costa Rica; Museu Biológico, Instituto Butantan, CEP 05503-900 São Paulo, SP, Brazil; Department of Ecology, Evolution, and Behavior, University of Minnesota, 1479 Gortner Ave., St Paul, MN 55108, USA; Department of Ecology, Evolution, and Behavior, University of Minnesota, 1479 Gortner Ave., St Paul, MN 55108, USA; Bell Museum, University of Minnesota, 1987 Upper Buford Circle, St. Paul, MN 55108, USA

**Keywords:** venom resistance, opossums, trench warfare, coevolution, convergent evolution, functional synthesis, ancestral-state reconstruction

## Abstract

Opossums in the tribe Didelphini are resistant to pit viper venoms and are hypothesized to be coevolving with venomous snakes. Specifically, a protein involved in blood clotting (von Willebrand factor [vWF] which is targeted by snake venom C-type lectins [CTLs]) has been found to undergo rapid adaptive evolution in Didelphini. Several unique amino acid changes in vWF could explain their resistance; however, experimental evidence that these changes disrupt binding to venom CTLs was lacking. Furthermore, without explicit testing of ancestral phenotypes to reveal the mode of evolution, the assertion that this system represents an example of coevolution rather than noncoevolutionary adaptation remains unsupported. Using expressed vWF proteins and purified venom CTLs, we quantified binding affinity for vWF proteins from all resistant taxa, their venom-sensitive relatives, and their ancestors. We show that CTL-resistant vWF is present in opossums outside clade Didelphini and likely across a wider swath of opossums (family Didelphidae) than previously thought. Ancestral reconstruction and in vitro testing of vWF phenotypes in a clade of rapidly evolving opossums reveal a pattern consistent with trench warfare coevolution between opossums and their venomous snake prey.

## Introduction

Ever since biologists and naturalists first recognized that patterns of adaptation and counter adaptation might explain reciprocal changes in phenotypes, coevolution has been a central idea in evolutionary biology (Priestley 1764; Darwin 1859; Müller 1873; Smith 1887; Andrews 1891; Von Ihering 1902). However, Janzen (1980) argued that though we observe coevolution as matched phenotypes in the present day, our inability to observe historical interactions among ancestral species makes it difficult to distinguish the role that coevolution played in shaping modern species interactions. For example, predators may be able to eat novel toxic prey because they already carry detoxifying abilities attained through some other process. Such preadaptations may make a new predator–prey interaction possible, but the adaptation did not result from trophic coevolution.

**Fig. 1.**
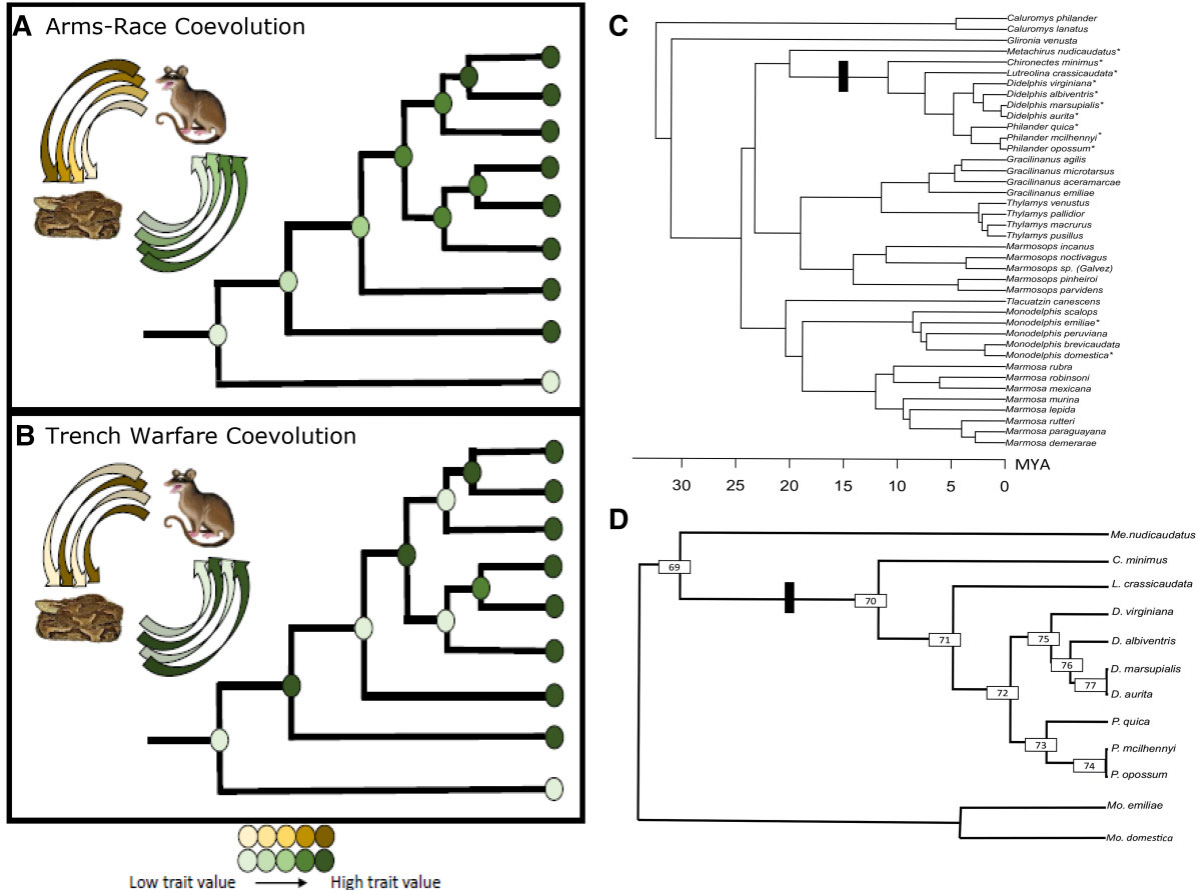
Predicted patterns of trait change under different models of coevolution (*A* and *B*) and the phylogenies used to test these predictions in this study (*C* and *D*). For each model, the evolution of interacting traits is indicated by colored arrows between the snake and opossum, with inner arrows representing ancestral phenotypes. The evolutionary history for this trait is mapped onto a phylogenetic tree to show the expected pattern of ancestral phenotype evolution given two different models of coevolution: (*A*) An expected pattern of phenotype evolution consistent with arms-race coevolution, where traits evolve directionally; (*B*) An expected pattern of phenotype evolution consistent with trench warfare coevolution, where traits toggle back and forth. These are not the only explanations for these patterns of trait evolution; however, they are consistent with what we would observe under these models of coevolution;. (*C*) Pruned time-calibrated tree from Jansa et al. (in prep.) used for reconstructing ancestral vWF sequences in this study. We refer to Didelphini (indicated with a black rectangle) as defined by Jansa and Voss (2011), with the updated species name *P. quica* used for *P. frentaus* of that study (Voss et al. 2018). Asterisks indicate opossum species whose vWF was selected for expression and binding assays. (*D*) The subclade of Didelphini + *Metachirus* pruned from this phylogeny and rooted with two species of *Monodelphis.* Numbered nodes are the ancestral vWF sequences that were estimated with ancestral-state reconstruction and expressed for binding assays.

Models of coevolutionary processes predict how ancestral phenotypes should have changed as a result of long-term reciprocal interactions between species. For example, arms-race coevolution—where antagonists evolve increasing defense/attack phenotypes, perpetually escalating their trait values—has been described as the dominant model of antagonistic coevolution (Dawkins and Krebs 1979; Abrams 1986; fig. 1*A*). An alternative model of coevolution, where phenotypes change by alternating their ability to match one another, has been described as “trench warfare” or “phenotype matching” coevolution (Ridenhour and Nuismer 2007; Gomulkiewicz et al. 2020), by virtue of its characteristic reciprocal retaliation (Axelrod and Hamilton 1981; fig. 1*B*). This model invokes a distinct process of cyclic fluctuating selection which generates traits that flip back and forth between trait values (Thompson 1994; Jokela et al. 2000). Ancestral phenotypes in this type of coevolution would instead be expected to be changing in a nonunidirectional fashion, toggling between trait values over time (Stahl et al. 1999; Brown and Tellier 2011).

These two coevolutionary models are difficult to distinguish without access to observations of ancestral interactions. Several approaches have been employed to observe ancestral phenotypes, including analyses of the fossil record (e.g., Kelley 1992; Grossnickle and Polly 2013), studies of experimental coevolution (Brockhurst and Kostella 2013), and engineering ancestral proteins (Golding and Dean 1998). This latter approach—sometimes termed the “functional synthesis”— examines the plausibility of hypothesized evolutionary scenarios by testing the function of ancestral proteins (Golding and Dean 1998). Models of sequence evolution are used to detect selection and reconstruct ancestral sequences, subsequently in vitro expression and functional assays are used to empirically test hypotheses of adaptive function in extant and ancestral proteins. Studies applying this approach have provided important new insights into the relative roles that constraint, epistasis, and permissive mutations play in molecular evolution (Dean and Thornton 2007; Bridgham et al. 2009; Harms and Thornton 2013). However, studies employing the functional synthesis have focused on adaptive evolution to abiotic selection pressures and have yet to be used in studies of coevolving proteins.

Ideally, a functional synthesis approach would be applied to both proteins involved in a coevolutionary interaction, but this goal has remained elusive due to the difficulty of reconstructing the evolutionary history of both participants. Nevertheless, evolutionary rate analysis and empirical ancestral phenotype data for just one of the interacting partners can still address the predictions made by different models of coevolution (fig. 1*A* and *B*). For example, if interacting proteins are not coevolving, but rather are preadapted for their current interaction, we should see no phenotypic changes across the phylogeny and would expect the gene to be evolving at low rates consistent with purifying selection. If a phenotype is a result of a single ancient adaptive event, but not an ongoing coevolutionary interaction, we might expect a single phenotypic shift early in the clade’s history, perhaps accompanied by a high branch-specific substitution rate in the gene. Subsequently, we would expect substitution rates to slow as phenotypic stasis is maintained. In the case of venom resistance, if either of these scenarios of preadaptation is true, we would expect venom resistance to be maintained throughout evolutionary history (e.g., all nodes in fig. 1*A* and *B* to exhibit venom resistance from the base of the tree onwards). This would also be the most parsimonious trait reconstruction assuming all descendent taxa are venom resistant.

Alternatively, if the protein shows evidence of rapid positive selection in the clade of interest—consistent with expectations of ongoing coevolution—then different patterns of ancestral protein phenotype evolution can distinguish either arms-race coevolution (fig. 1*A*) or trench warfare coevolution (fig. 1*B*). Although rapid positive selection is expected in a coevolutionary dynamic, it may also signal ongoing adaptive evolution such as an adaptive radiation or rapid environmental change. Thus, examination of both the ancestral and contemporary phenotypes and genotypes is necessary to distinguish between these scenarios.

We apply this approach to examine the evolution of a blood-clotting protein (von Willebrand factor [vWF]) that has apparently evolved to confer resistance to snake venom C-type lectins (CTLs) in some species of South American opossums (Clade: Didelphini; Jansa and Voss 2011; Drabeck et al. 2020; fig. 1*C* and *D*). Botrocetin, a vWF-targeting CTL, disrupts normal blood coagulation by first binding tightly to the A1 domain of vWF, then inducing vWF to bind the platelet-associated glycoprotein GP1Bɑ, and finally binding to GP1Bɑ itself (Fukuda et al. 2005). This tri-molecular complex (botrocetin–vWF–GP1Bɑ) is the ultimate source of hemostatic disruption, preventing vWF and platelets from functioning normally (Maita et al. 2003). A second venom CTL, aspercetin, has been shown to induce thrombocytopenia via vWF, though the specific vWF domain(s) and platelet-binding site it targets is unknown (Rucavado et al. 2001). Both botrocetin and aspercetin are derived from South American vipers (*Bothrops jararaca* and *B. asper*, respectively) that are sympatric with and known prey items of venom-resistant opossums (Oliveira and Santori 1999). Prior research (Jansa and Voss 2011) discovered that vWF is evolving under accelerated positive selection in a group of opossums known to be resistant to snake venoms (Didelphini). Further work has shown that blood from several opossums do not produce a coagulation response to venom CTLs (Drabeck et al. 2020). Together, this suggests that the two proteins involved in this interaction—vWF and its snake venom CTL agonist, botrocetin—might be involved in a coevolutionary arms race.

To test these predictions of coevolution, we first require evidence that botrocetin (and other vWF-binding venom CTLs) can no longer bind vWF to induce hemostatic disruption in resistant opossum species. We next require an understanding of how ancestral vWF proteins interact with these venom agonists. In this study, to empirically assess binding affinity between these two proteins, we measured binding kinetics in vitro and calculated the equilibrium dissociation constant (*K_D_*) as well as association (*K*_on_) and dissociation (*K*_off_) rate constants. For modern species of opossum, we expressed vWF from several opossum species, including venom-resistant members of Didelphini (fig. 1*C* and *D*), as well as two species of *Monodelphis* that are assumed to be venom sensitive as they are common prey of *Bothrops* (Voss 2013; Drabeck et al. 2020). We also included human vWF to represent a species which is venom sensitive at the organismal level, binds to vWF in assays, and is known to function well as a heterologously expressed domain. For assays of ancestral protein function, we inferred ancestral opossum vWF sequences and expressed these vWF proteins in vitro. We assayed binding affinity of modern and ancestral vWF proteins for four isolated venom CTLs (two isoforms of botrocetin, aspercetin, and bitiscetin from an African viper). Together, these data address the contemporary interactions between vWF and venom agonists, and allow us to assess whether evolution of this protein in opossums is consistent with a coevolutionary process, and if so, which dynamic—arms-race or trench warfare coevolution—best describes this interaction.

## Results

### Binding Assays from Extant Taxa

**Fig. 2.**
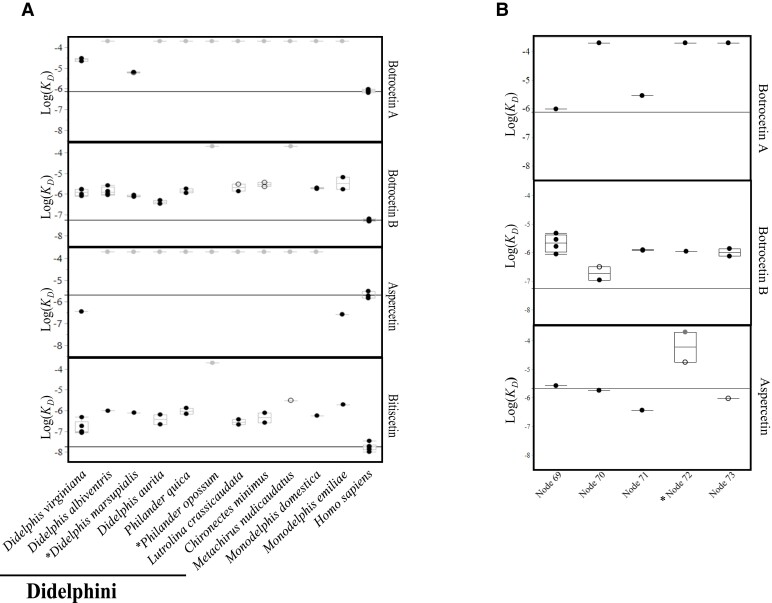
(*A*) Binding affinity (log_10_*K_D_*) of vWF from extant species for four venom proteins (two isoforms of botrocetin, aspercetin, and biticetin). vWF proteins were expressed from the opossum species shown along the *X*-axis; the bold black line indicates species within Didelphini previously proposed to be resistant to the venom protein botrocetin. Lower log_10_*K_D_* values represent stronger binding; the horizontal line across each plot shows the average log_10_*K_D_* value for human; values above this line represent relatively weaker binding between vWF and the venom agonist. Assays resulting in no detectable binding were given an arbitrarily low binding affinity (log_10_*K_D_* = −4) and are indicated by gray circles. Estimated *K_D_* values with associated low *R*^2^ values (0.91–0.95) are indicated by empty circles; those with *R*^2^ values of 0.96 or greater are indicated by black circles. Standard quantile box plots show a line at the median and enclose the 25th and 50th quantiles of the distribution. (*B*) Binding affinity (log_10_*K_D_*) of ancestral vWF proteins for two isoforms of botrocetin and aspercetin. Node numbers refer to the ancestral nodes labeled in figure 1*D*. Asterisks denote that the following sets have identical vWF protein sequences: (nodes 72 and 75); (*D. marsupialis* and node 76); (*P. mcilhennyi*, *P. opossum*, and node 74).

There was no evidence of binding between botrocetin (isoform A, hereafter botrocetin A) and vWF for 9 of the 11 extant opossum species tested; for the remaining two species (*Didelphis virginiana* and *D. marsupialis*) binding affinity was reduced at least eight-fold compared with human vWF (fig. 2*A*). For botrocetin (isoform B, hereafter botrocetin B), binding affinity for vWF was reduced at least eight-fold for all opossum species compared with human, and one species (*Metachirus nudicaudatus*) showed no evidence of binding (fig. 2*A*; lack of binding for *Philander opossum* in this assay is attributed to misfolded vWF, as discussed below). For Aspercetin, only two opossum species (*Mo. emiliae* and *D. virginiana*) showed binding between vWF and the venom agonist, both with higher affinity than human vWF (fig. 2*A*).

Misfolding of synthetic vWF proteins could cause false negatives in our assays if synthetic vWF does not bind botrocetin simply because the protein was incorrectly folded. Therefore, as a control for protein activity, we assayed the binding affinity of synthetic vWF with biticetin, which should bind active, synthetic vWF even if botrocetin does not (see Methods). Using this assay, our binding results suggest the presence of properly folded, active vWF for all species except *P. opossum*, which did not produce a binding curve between vWF and bitiscetin (fig. 2*A*).

### Binding Results for Ancestral vWF

**Fig. 3.**
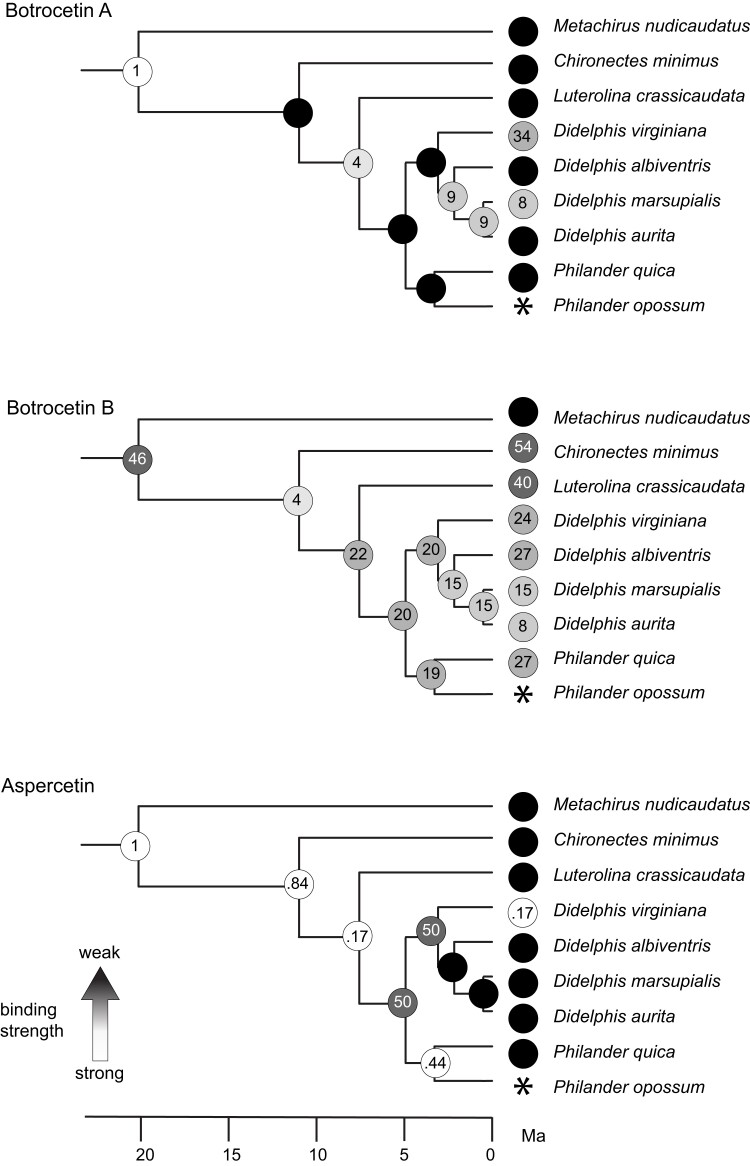
Time-scaled phylogenies of opossum species showing the relative loss of binding affinity between vWF and the venom agonist (two isoforms of botrocetin and aspercitin) for expressed modern (tip) and ancestral (node) vWF proteins. Binding loss is expressed as the ratio between the observed K_D_ value for the expressed protein and the K_D_ value from human vWF binding to the given venom agonist. Lower values (and lighter colors) represent stronger binding: values of 1 indicate that agonist binds the expressed vWF protein equally as well as it binds human vWF; values >1 indicate the multiple of observed binding loss relative to human (e.g., binding is 4–54 times lower than human vWF affinity for the agonist); values <1 indicate that the expressed vWF protein binds the agonist more strongly than it does human vWF; black circles with no values indicate no detectable binding between that vWF protein and the agonist. Binding affinity for *P. opossum* vWF is indicated with an asterisk as these values could not be accurately determined due to probable misfolding of this protein.

A total of five ancestral sequences were reconstructed representing eight nodes in the didelphid phylogeny (fig. 1*D*; nodes 73 and 74 have identical amino acid sequences as have nodes 76, 77, and *D. marsupialis*; supplementary file S2, Supplementary Material online). Kinetic data for ancestral opossum vWF proteins were collected for botrocetin A, botrocetin B, and aspercetin (fig. 2*B*) and are shown in a phylogenetic context in figure 3. For botrocetin A, binding affinity for the oldest vWF protein tested (at node 69 which defines the split between *Metachirus* and Didelphini; fig. 3) is comparable with human vWF; sequences occurring at more recent splits show either four to nine-fold reduced binding compared with human vWF or have lost the ability to bind vWF completely (figs. 2*B* and 3). For botrocetin B, all ancestral proteins tested have 15- to 46-fold reduced binding compared with human vWF, except the node subtending Didelphini, which has only four-fold loss of binding (figs. 2*B* and 3). For aspercetin, most ancestral opossum vWF proteins either bind as well as or better than human vWF; the exceptions are the sequences defining the split between *Didelphis* and *Philander* (fig. 3), and ancestral nodes within *Didelphis*, which have at least a 50-fold reduction of binding compared with human vWF.

### Functional Interpretation of Binding Results

For most species, we lack data from functional assays that would allow us to determine the level of reduced binding affinity that would result in a nonfunctional venom; however, we do know that botrocetin B has an eight-fold lower affinity for vWF from *D. aurita* than for human vWF, and that platelets from this opossum species fail to aggregate when exposed to this venom protein (Matsushita and Sadler 1995). Therefore, we infer that an eight-fold loss in affinity between vWF and venom molecules is sufficient to abolish an aggregation response to venom CTLs.

### Influence of K_on_ and K_off_

Weak binding between a protein and a target may result from a reduction in the rate at which a protein binds on to its target (association, or on-rates) or an increase in the rate at which it falls off its target (dissociation, or off-rates). Both processes can change overall binding (*K_D_*) but are often the result of distinct biochemical changes (mutations). To determine if reduced binding affinities of vWF are attributable to changes in association (on-rates) or dissociation (off-rates), we first grouped our kinetic data from all protein–protein interactions into two classes: binders and nonbinders. Nonbinders were defined as protein–protein interactions that showed at least an eight-fold loss of binding between the toxin and vWF compared with human (*n* = 44); all other interactions were classed as binders (*n* = 31). Binders did not have significantly faster on-rates than nonbinders (*P* = 0.65, *t* = −0.45, df = 2). However, binders did have significantly slower off-rates compared with nonbinders (*P* = 0.038, *t* = −2.10, df = 2).

## Discussion

Changes in ancestral binding affinities observed here are not predicted from simple phylogenetic trait reconstructions based on the binding affinities observed in modern taxa. Both isoforms of botrocetin have low affinity for vWF among extant species of didelphines, suggesting that all ancestral sequences in this clade should have reduced affinity as well (if a simple model of character state reconstruction applies, supplementary fig. S3, Supplementary Material online). Similarly, aspercetin shows affinity only for vWF from *D. virginiana* among modern taxa, suggesting that aspercetin binding is uniquely evolved in this species and so should also be absent among ancestral sequences. The fact that we find variation in binding affinities among our ancestral proteins where none is predicted by ancestral trait reconstruction reinforces the power of a functional synthetic approach in testing hypotheses of molecular evolution, especially for rapidly evolving proteins over relatively long timescales. In this case, the trait “binding affinity” is affected by several amino acid changes in the vWF molecule. Thus, simply reconstructing binding affinity does not capture the underlying complexity of molecular changes that affect this trait or take into account that rapid positive selection has been at work across this clade.

The three venom toxins show different patterns of affinity for the same ancestral opossum vWF proteins, suggesting that each has experienced a different history of coevolution with vWF (fig. 3). In no case do we see the unidirectional changes for reduced binding affinity expected of a coevolutionary arms race (fig. 1*A*). Rather, each of the venom CTLs shows a pattern of repeated increasing and decreasing binding affinities more consistent with a trench warfare model, in which traits toggle back and forth in response to a coevolving partner (fig. 1*B*). For example, botrocetin A loses its affinity for vWF along the relatively long branch separating *Metachirus* from the large-bodied opossums (fig. 3), but some of that affinity is regained in the ancestor of *Lutreolina*, *Didelphis*, and *Philander*, only to be lost again in its descendants. Fewer changes characterize the evolution of vWF’s affinity for botrocetin B, which starts low, increases along the same long-branch separating *Metachirus* from the other opossums, and then is lost again (fig. 3). Finally, aspercetin retains its affinity for vWF throughout the early evolution of this clade, is lost in the ancestor of *Didelphis* and *Philander*, and is regained twice independently, once in *D. virginiana* and once in the ancestor of *Philander* species (fig. 3). Interestingly, two different sources (isoforms) of botrocetin (A and B) show different patterns of resistance and may represent a functional polymorphism as is predicted in trench warfare coevolution (Stahl et al. 1999; Woolhouse et al. 2002).

Our results also show that resistance to venom CTLs is more widespread among extant opossums than previously suspected. Although we had expected to find reduced binding affinities between venom CTLs and vWFs from the large-bodied venom-resistant species in Didelphini, we also found reduced binding affinities for vWFs from two species of *Monodelphis*—small-bodied opossums that are not known to be venom resistant—as well as from *Metachirus*, which has shown venom sensitivity in some assays (Perales et al. 1994). Superficially, these observations are consistent with a scenario of preadaptation, in which having a vWF molecule that does not bind venom CTLs is simply a trait inherited by venom-resistant taxa from their ancestor. Yet, changes in the binding affinities among ancestral large-bodied opossum vWFs, together with the accelerated rates of vWF sequence evolution in Didelphini along branches leading to some species of *Monodelphis* and *Marmosa* (Jansa and Voss 2011), implicate broader coevolutionary processes.

Drabeck et al. (2020) showed that *Mo. domestica* exhibits physiological resistance to botrocetin measured as a lack of platelet aggregation response. Kinetics data reported here from botrocetin are consistent with those results, and further suggest that *Me. nudicaudatus* and *Mo. emiliae* likely also exhibit physiological resistance to botrocetins. If we use the eight-fold loss of binding seen in *D. aurita* as a threshold for protection for all venom CTLs, we predict that all species tested in this study are resistant to all CTLs except *D. virginiana* and *Mo. emiliae* for aspercetin. The general agreement of physiological data from previous work and kinetics data reported here strongly suggest that all opossums tested in this work either within or outside Didelphini enjoy physiological resistance to multiple isoforms of botrocetin (Matsushita and Sadler 1995; Drabeck et al. 2020).

Though this result was expected for species within Didelphini (particularly species for which organismal venom resistance has been demonstrated), it was not expected for species outside this group. Previous work has reported *Me. nudicaudatus* as venom sensitive; however, this assertion relies on a single study in which two individuals died after an injected dose of *B. jararaca* venom (Perales et al. 1994). Though this outcome certainly indicates that *Me. nudicaudatus* is sensitive to whole venom; this species may still have vWF that is resistant to venom CTLs. Whereas resistant vWF alone would likely not confer organismal resistance to whole venom, it may suggest that partial venom resistance for *B. jararaca* venom, or the venom of a closely related viper, may be important in this species. Venom and venom resistance are complex traits, of which vWF is likely to be an important component. Combined with other components of resistance (e.g., metalloproteinase inhibitors, phospholipase inhibitors), this trait likely contributes significantly to venom resistance.

Although the ecological selection pressure driving venom resistance within Didelphini appears to be a dietary adaptation that enabled large-bodied opossums to exploit venomous snakes as a food source (Jansa and Voss 2011; Voss 2013), opossums outside this clade are smaller bodied and more likely prey of *Bothrops* spp. (Voss 2013). Whereas *Mo. domestica* has been reported to eat snakes, specific species in their diet have not been noted, and known instances of predation on *Monodelphis* spp. by *Bothrops* spp. are more common (Voss 2013). Other species thought to have evolved venom resistance as a dietary adaptation include mammals that exhibit exceptionally strong and generalized antivenom competence (e.g., hedgehogs, mongoose, honey badgers, grasshopper mice; Barchan et al. 1995; Rowe et al. 2013). In contrast, species known to have evolved venom resistance as a predator defense generally show weaker venom resistance that is highly variable by species and geographic range (Holding et al. 2016; Pomento et al. 2016). Although CTL-resistant vWF does not by itself appear to confer resistance to whole venom, it may be indicative of selection pressures for partial or local resistance for species outside Didelphini which experience heavy predation by *Bothrops* spp. Repeated convergent evolution of CTL-binding loss reported here, as well as elevated rates of vWF evolution along branches leading to species of *Monodelphis* and *Marmosa* (Jansa and Voss, 2011), suggests that evolution for venom-resistant vWF may be important for small-bodied opossums as well. These and previous data (Drabeck et al. 2020) demand further investigation of venom resistance across Didelphidae.

**Fig. 4.**
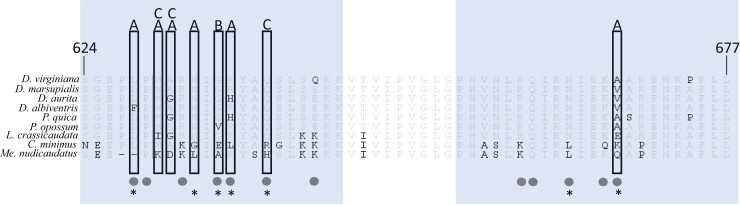
Alignment of vWF protein sequences for species of Didelphini + *Metachirus*. Sites associated with multiple independent instances of greater than ten-fold binding loss between the expressed vWF protein and a venom agonist are enclosed in boxes, and the venom agonist is denoted above the box, as follows: A—botrocetin A; B—botrocetin B; C—aspercetin. Known botrocetin binding sites are marked with a circle, and sites evolving under positive selection per Jansa and Voss (2011), are denoted with an asterisk. Regions 624–677 are shown as it encompasses the two botrocetin binding pockets (624–645), and (655–677) and are in shaded boxes. Majority amino acids are in gray, and variants are denoted in black.

Nearly half of the amino acid sites in the CTL-binding region of vWF exhibit changes in Didelphini (fig. 4); of these, seven show evidence of positive selection (Jansa and Voss 2011). Based on extant sequences alone, it is difficult to infer which of these rapidly evolving sites contribute to changes in the binding affinities for venom CTLs. However, the addition of functional data from reconstructed ancestral proteins allows us to identify amino acid changes on several branches that show repeated instances of greater than ten-fold changes in binding affinities (table 1).

**Table 1.** Sites which are Associated with Greater than Ten-Fold Binding Loss At Least Twice (on two branches independently) within Didelphini + *Me. nudicaudata*.

Site	Change	Branch	Binding loss for
628*	Deletion	Node 69→*Me. nudicaudatus*	Botrocetin A, aspercetin
	L→F	Node 76→*D. albiventus*	Botrocetin A
630	M→I	Node 71→ *Lutreolina crassicaudata*	Botrocetin A, aspercetin
	M→K	Node 69→*Me. nudicaudatus*	Botrocetin A, aspercetin
631*	A→G	Node 71→ *L. crassicaudata*	Botrocetin A, aspercetin
	A→G	Node 73→*P. quica*	Botrocetin A, aspercetin
	A→D	Node 69→ *Me. nudicaudatus*	Botrocetin A, aspercetin
633*	S→L	Node 69→*Me. nudicaudatus*	Botrocetin A
	S→G	Node 69→node 70	Botrocetin A
635*	D→E	Node 70→*C. minimus*	Botrocetin B, aspercetin
	D→G	Node 70→ node 71	Botrocetin B, aspercetin
636*	R→H	Node 77→ *D. aurita*	Botrocetin A
	R→L	Node 69→node 70	Botrocetin A
639*	Q→R	Node 70→ *C. minimus*	Aspercetin
	Q→H	Node 69→ *Me. nudicaudatus*	Aspercetin
**668**	Q→K	Node 69→node 71	Botrocetin A
	E→A	Node 71→ node 72	Botrocetin A

Sites in bold are known botrocetin binding sites (Fukuda et al. 2005), and sites with asterisks are evolving under positive selection per Jansa and Voss (2011). Changes with the exact same amino acid changes are colored in gray.

Notably, not all of these changes agree with predictions from prior site-directed mutagenesis studies based on human vWF. Previous work found four mutations in vWF (R629A, R632A, R636A, and K667A), each of which reduced human vWF affinity for botrocetin by at least 60% (Matsushita et al. 2000). Of these, site 629 is a proline residue across all opossum species; site 632 is Arg among most, but Lys in *Chironectes* and *Metachirus*. For human vWF, site 636 required an Arg residue and site 667 required Lys to successfully bind botrocetin. Among venom-resistant opossums, site 636 shows evidence of rapid evolution and repeated loss of the ancestral Arg residue in *D. aurita*, *P. quica*, and *C. minimus*, all of which have lost or reduced botrocetin binding ability according to our assays. However, opossum species that have retained an Arg in this position also show reduced affinity for both isoforms of botrocetin, and ancestral sequences with 636^Arg^ show variation in botrocetin binding ability. Similarly, site 667 has a Lys residue in most species of opossums (only *Chironectes* differs with 667^Gln^) and binding affinity varies among both modern and ancestral sequences with 667^Lys^. Additional site-directed mutagenesis studies showed that tandem mutations at the lysine stretch 642–645 disrupt botrocetin binding for human vWF (Matsushita and Sadler 1995; Matsushita et al. 2000). Whereas we do see the loss of two of these lysines from node 71 to node 72 in Didelphini, this branch only shows loss of binding for botrocetin A and aspercetin. Thus, changes in opossum vWF affinities for CTLs appear to be more complex than the single-point changes reducing binding between human vWF and botrocetin.

Our results add to previous evidence that opossums and pit vipers may be coevolving, the pattern recovered here could also be explained by intermittent selection for binding loss where a very high physiological cost drives reversals when selection against toxins is not present. Whereas the physiological consequences of the observed mutations on vWF in opossums are unknown, it is possible that they may interfere with efficient binding of vWF to collagen, platelets, or both. Further physiological investigation is needed to elucidate the potential for physiological tradeoffs of these venom-resistance mutations. This pattern may also be consistent with opossums expanding their diet to multiple species of *Bothrops* with differing CTLs, essentially mimicking reciprocal venom changes via prey switches. CTLs among viperids are diverse and, like most venom proteins, exist in nonidentical tandem arrays of 10–50 copies. Although this work has focused on the three currently known vWF-binding CTLs, there are certainly more undescribed vWF and other integrin-binding CTLs which have yet to be described and would likely contribute to furthering our understanding of this coevolutionary dynamic.

Ultimately, further work reconstructing the history of venom vWF-binding CTLs and testing ancestral botrocetins in vitro would be necessary to confirm the assertions that venom CTLs are in fact evolving reciprocally with opossum vWF. With recent advances in snake venom genomics and venomics (Amazonas et al. 2018; Gibbs et al. 2018) as well as recent work in developing an in vitro expression system for botrocetin (Matsui et al. 2017), this system in uniquely poised to characterize reciprocal functional and molecular changes in ancestors for *both* interacting partners in a natural system; a goal that has remained elusive in coevolutionary work thus far (Lovell and Robertson 2010; Scanlan et al. 2011).

## Materials and Methods

### vWF Sequencing

To express opossum vWF-A1 protein, the entire sequence of the region used for previous expression studies on human vWF (residues 475–709) was required (Cruz et al. 2000; numbering scheme see Maita et al. 2003). vWF sequences from species within and outside Didelphini were used from a previous study (Jansa and Voss 2011); however, the N-terminal region (∼207 bp) of the vWF-A1 region (residues 475–543) was missing from these individuals. We therefore sequenced this upstream region and assembled it with existing sequences using a ∼50 bp overlapping region. The vWF-A1 region was newly sequenced for three species (*P. mcilhennyi*, *D. marsupialis*, and *D. aurita*). Permission for the capture and sampling of *D. aurita* was granted by SISBIO (permit #64934-1). Sequences for human and *Mo. domestica* were downloaded from whole genome sequences available from NCBI (supplementary table S1, Supplementary Material online). Amplification of the target regions were performed with polymerase chain reactions (PCRs) as previously described (see supplementary methods and supplementary table S1, Supplementary Material online for primers; Jansa and Voss 2011).

### Ancestral Sequence Reconstruction

We used a codon model (CODEML) for maximum likelihood reconstruction of ancestral sequences as implemented in PAMLx version 1.3.1, PAML version 4.9 (Yang 2006; Jansa et al. 2014; Paradis and Schliep 2018). These analyses require two components: (1) vWF sequences from extant taxa, and (2) a tree with branch lengths for all species included (Yang 2007; Xu and Yang 2013). Complete vWF sequences described above (supplementary table S1, Supplementary Material online excluding human and *D. aurita*) as well as 28 additional opossum vWF DNA sequences (covering residues 543–709) from a previous study (Jansa and Voss 2011) were used to make a 39-taxon vWF alignment in Geneious version 7.1.8 using MUSCLE (Edgar 2004; supplementary file S3, Supplementary Material online). (2) An unpublished phylogeny (Jansa et al., in prep.) resulting from Bayesian analysis of six genes (CYTB, BRCA, IRBP, and three nuclear introns) and 121 taxa with the same calibration points as (Jansa et al. 2014) was pruned down to this same set of 39 species using R-package *ape* (supplementary file 1, Supplementary Material online; Jansa et al. 2014).

We used this alignment and phylogeny to compare models of selection in PAML version 4.9 (Yang 2006, 2007; Xu and Yang 2013). Specifically, we compared the distribution models M8 and M7 with test whether a model (M8) that allows for positive selection (d*N*/d*S* > 1) was a significantly better fit than a model that only allows for neutral or purifying selection (M7). The relative fit of the two models was measured by the natural log of the likelihood ratio (lnΛ), where −2[lnΛ] under the null hypothesis was assumed to follow a χ^2^ distribution with two degree of freedom (Yang 2006). We inferred ancestral sequences for nodes across the entire phylogeny using the best-fit model M8.

Nine of these ancestral sequences were selected for expression in *Escherichia coli*. These included all nodes within Didelphini as well as the most recent common ancestor of Didelphini and *Me. nuducadatus* (fig. 1*D*). We used parsimony to reconstruct presence or absence of a deletion at the N-terminal end of ancestral sequences as well as a single deletion event mid-sequence on the branch leading to *Me. nudicaudatus* (supplementary fig. S1, Supplementary Material online).

### Gene Synthesis

Extant and ancestral vWF sequences were edited in Geneious version 7.1.8 to contain N-terminal leader BamHI (5′-CACGGTAGC-3′) and C-terminal HinDIII (5′-TAACAAGCTTAA-3′) cut sites, optimized for *E. coli* codon usage, and submitted for gene synthesis by GenScript (Piscataway, NJ, USA). Several sequences were identical at the protein level, and therefore synthesized only once. A complete list of unique extant and ancestral constructs can be found in supplementary file S2, Supplementary Material online. Constructs were received in a pUC57 backbone plasmid (Tai et al. 2013).

### Vector Construction

A pQE9 plasmid vector containing an N-terminal 6-Histidine tagged Human vWF-A1 fragment (residues 475–710) was kindly provided by Dr Miguel Cruz (Baylor College of Medicine). This plasmid was used for expression of human vWF and subsequently modified for expression of extant and ancestral opossum vWF (Cruz et al. 2000; supplementary fig. 2, Supplementary Material online). Extant and ancestral opossum sequences were excised from pUC57 via BamHI/HinDIII restriction digest and ligated into purified pQE9 from which human vWF had been excised by the same digestion. pQE9 at this site has been engineered such that a ligated product will be placed directly downstream from a start codon and a 6-Histidine tag (supplementary fig. 2, Supplementary Material online). For efficiency, a pQE9 plasmid for *D. aurita* was constructed by site-directed mutagenesis PCR on the *D. marsupialis* pQE9 construct. Two protein coding differences at the binding site exist between these two species and were altered accordingly via Golden Gate Assembly (Engler et al. 2008). A single protein coding difference, four residues from the N-terminus, and outside the botrocetin binding site was not altered. See supplementary methods, Supplementary Material online for a detailed plasmid isolation protocol.

### vWF Expression and Purification

To produce active vWF protein capable of binding to venom CTLs in vitro, plasmid constructs were overexpressed and purified. Overexpression and purification was carried out as described previously (Cruz et al. 2000), with the following modifications. After addition of DNase, cell lysate was subjected to 3 min of sonication with a 6 mm microtip (Q500; Qsonica; 5 s on, 5 s off) with a 50% duty cycle. Purification was carried out as described previously (Cruz et al. 2000). Sonicated lysate was briefly centrifuged and supernatant was discarded. Remaining inclusion body proteins were unfolded in 6 M Guanidium-HCL, filtered, and refolded by dilution. Diluent containing refolded protein was applied to a 15-ml nickel column (GE Life Sciences) overnight and fractions containing vWF were eluted at 350 mM imidazole. Whereas (Cruz et al. 2000) using a heparin column for further purification, the heparin binding site in human vWF-A1 is not conserved in opossums; thus, we used an altered protocol to ensure a folded monomeric product. Once eluted off nickel column, fractions were dialyzed into 100 mM Tris-HCl, 400 mM NaCl, 1 mM EDTA, pH 7.4, and applied to a 10-ml Thiopropyl Sepharose 6B column (GE Life Sciences). Folded protein was collected in the flow-through (Miura et al. 2000). Flow-through was then dialyzed in 10 mM Tris-HCL, 100 mM NaCl pH 7.5 at 4 °C overnight, and concentrated to 5 ml or less via ultrafiltration in a Vivispin 15 10,000 molecular weight cutoff spin column (Sartorius, Goettingen, Germany). This concentrated sample was applied to a size exclusion column (16/600, 200 pg Superdex; GE Life Sciences), and the final peak was collected as monomeric-folded vWF-A1. Product was concentrated via ultrafiltration, checked for size and purity on a Tris-glycine gel, quantified by absorbance at 280 nm (NanoDrop model 2000; Thermo-Scientific), aliquoted, and frozen at −80 until use. Purification was done for each protein separately on an ATKA FPLC (GE Healthcare). Purified vWF protein for each species/node was stored in separate freezer boxes and labeled individually throughout to avoid cross-contamination.

Sequences inferred from ancestral-state reconstruction were transformed and overexpressed months apart from each other (with the exception of nodes 72 and 73 which were expressed on the same day) to reduce the possibility of samples being accidentally switched or contaminated. As a control, vWFs from *Mo. domestica* and *P. quica* were expressed twice, months apart from one another. Resulting affinity measurements were comparable (supplementary table S1, Supplementary Material online).

### Venom Purification

Aspercetin was purified as previously described (Xu and Yang 2013), reconstituted, and dialyzed into TBS (25 mM Tris-HCL, 150 nM NaCl pH 7.4). Two isoforms of botrocetin were used. One (“botrocetin A”) was kindly gifted by Dr Robert Andrews, Monash University, and was among the batches used for the first studies of botrocetin structure and function (e.g., Dong et al. 2001). A second (botrocetin B) was purified from *B. jararaca* venom as previously described (Drabeck et al. 2020). Both samples botrocetin A and botrocetin B were the same isoforms used in Drabeck et al. (2020). Bitiscetin was purified as previously described and used to confirm that *E. coli* expressed and refolded vWF-A1 was capable of binding a venom CTL in the assay conditions described below (Hamako et al. 1996; Drabeck et al. 2020).

### Binding Assays

Binding affinities for vWF and venom proteins were measured using a white light biolayer interferometry system, BLItz (ForteBio; Pall Corporation). Experimental design was optimized according to manufacturer’s recommendations (Sultana and Lee 2015). The BLItz design was chosen as it allows for data collection of each binding interaction with extremely small volumes of venom protein (4 μl/curve), and therefore extended the ability to collect data given very limited sample volumes. Each binding curve was obtained by first immobilizing vWF (the ligand) to a surface (both HS1K [antihistidine antibody] and NiNTA [Nickel chip] tips were used) and then exposing it to a venom CTL (the analyte). Association and dissociation of the analyte is observed in nearly continuous time, resulting in a binding curve (association and dissociation). Venom proteins (botrocetin A, botrocetin B, aspercetin, and bitiscetin) and vWF-A1 aliquots were stored at −80 °C and thawed on ice until immediately prior to use. Detailed BLItz protocols can be found in supplementary methods, Supplementary Material online.

### Data Analysis

Raw kinetics data were globally fit to a 1:1 binding model using the BLItz Pro software v1.2.1.3. For a full description of curve fitting and affinity calculations see supplementary methods, Supplementary Material online. Curve sets were optimized by local fitting first, and curves which both had extremely low *R*^2^ values and significantly changed the globally calculated *K_D_* and *R*^2^ were excluded per recommendation by the manufacturer (ForteBio; Pall Corporation). All resulting kinetics estimates were extrapolated from a minimum of three curves (concentrations of venom CTLs). All data estimated from the global fit for each curve set (vWF–venom protein pair) were exported into a .CSV file for further analysis.

Binding is reported relative to human binding rather than relative to a venom-sensitive opossum, as all opossums tested appeared to have significant loss of binding, as well as accelerated rates of vWF evolution despite being previously assumed to be venom sensitive. Human vWF is known to bind botrocetin, to aggregate platelets in physiological assays, and to cause organism-wide physiological response to venom CTLs. It is therefore used as our benchmark of venom CTL susceptibility.

Summary statistics and subsequent analyses of binding curve data extracted from BLItz software were calculated in JMP Pro version 14.0.0 (SAS Institute). Variance in *K_D_* is right skewed as larger numbers (higher *K_D_*) will have associated larger error. A standard least squares regression was used to assess the assumption that error (variance) will increase with weaker binding, and identify data points that exhibited variance which was higher than expected (outside a 95% confidence interval), above the regression curve. These data points are further addressed in the results. Because of the skewness of this data, *K_D_* was log_10_ transformed for subsequent analyses. Log-transformed *K_D_* was also regressed against variance to determine resolution of skewness. As each exported assay has an associated *K_D_* and *R*^2^ value, *R*^2^ was regressed with *K_D_* to determine if goodness of fit (*R*^2^) reduced with increasing *K_D_*, which would indicate systemic poor fit for high *K_D_* values.

To test how differences in on-rates (*K*_on_) and off-rates (*K*_off_) influence binding loss, data were grouped into binders (1–8× human binding loss), and nonbinders (>8× human binding loss). We interpret that an eight-fold loss in binding affinity between vWF and venom molecules would be enough to abolish an aggregation in response to venom CTL, as *D. aurita* vWF has an eight-fold lower affinity for botrocetin and live platelets in platelet-rich plasma from this species fail to aggregate in the presence of botrocetin (Drabeck et al. 2020). Nonparametric Wilcoxon tests were used on log-transformed *K*_on_ and *K*_off_ to assess differences between these groups (binders and nonbinders).

### Parsimony Reconstruction of Binding Phenotypes

To assess whether inferred ancestral binding affinity differed from actual measured affinity of expressed proteins, we estimated the expected binding affinity based on extant phenotypes using models of trait change applied to the didelphid phylogeny. Measured binding affinity of opossum vWF from biophysical assays was divided by human binding affinity for the same protein to generate a measure of relative binding capacity for each species. Because relative binding spans values from 0.17 to 10,000×, binding loss was coded as a discrete, ordered state by binning relative binding loss by increments of 5× binding loss: (1) 0–5×, (2) 6–10×, (3) 11–15×, (4) 16–20×, (5) >20×, and (6) no detectable binding Parsimony and maximum likelihood reconstruction was used to infer ancestral binding affinities on the same phylogeny as described above; all methods of inference yielded similar results; we present reconstructions from squared change parsimony as inferred in the R-package *castor* (function *asr_squared_change_parsimony*, version 1.3.6) with unweighted transition costs (*weighted* = *FALSE*; Maddison 1991; Louca and Doebeli 2017; Voss et al. 2018).

## Supplementary Material

Supplementary data are available at *Molecular Biology and Evolution* online.

## Supplementary Material

msac140_Supplementary_Data

## Data Availability

All raw kinetics data, constructs, trees, and detailed methods for this work are included in the supplementary materials.
