# Supplementary material for: Ancestrally Reconstructed von Willebrand Factor Reveals Evidence for Trench Warfare Coevolution between Opossums and Pit Vipers
Source: Mol Biol Evol. 2022 Jun 20;39(7):msac140. doi: 10.1093/molbev/msac140 (PMC9255381; doi:10.1093/molbev/msac140)
Supplement: msac140_Supplementary_Data [file msac140_supplementary_data.zip › Sup Figure 1.pdf]

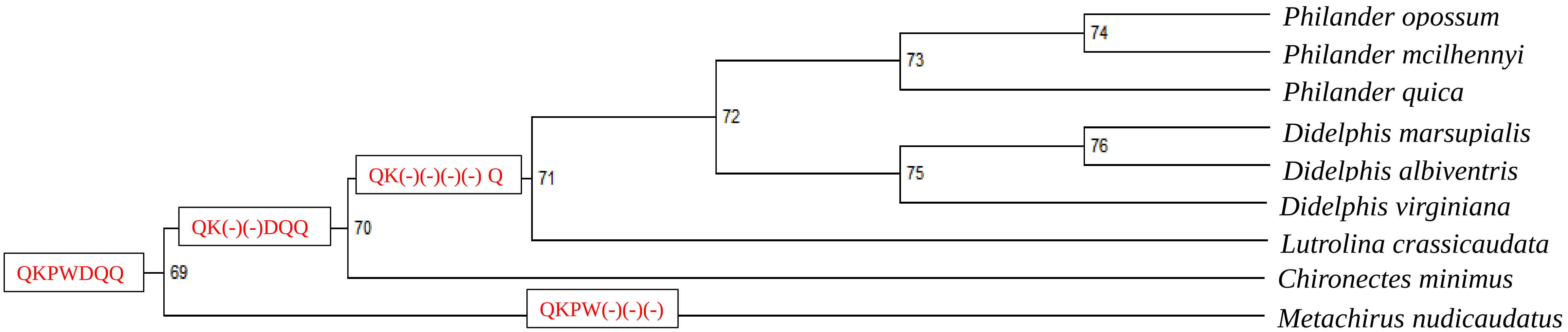

Supplementary Figure 1- Parsimony reconstruction of N-terminal sequence gaps for clade Didelphini. Didelphini is defined as the clade beginning at node 70. Node labels are arbitrarily designated by the program PAML and are used for reference in ancestral reconstruction. Ancestral reconstruction identifies nodes/species identical at the proteins level as nodes (72,75), (76, D. marsupialis), (74, P. mcilhennyi, P.opossum). An additional node generated by the addition of D. aurita as sister to D. marsupialis (not shown) is identical to D. marsupialis. Ancestral state reconstruction was originally conducted using an alignment of 39 species based on sequence availability of vWF (Supplementary File 3). Sequences of Didelphis aurita were subsequently generated for expression and added to the alignment (Supplementary File 4). Re-estimation of ancestral sequences with the addition of Didelphis aurita resulted in one amino change in one ancestor (node 70). This alternative ancestral sequence was not subsequently cloned and used for in-vitro expression.
