# Supplementary material for: Ancestrally Reconstructed von Willebrand Factor Reveals Evidence for Trench Warfare Coevolution between Opossums and Pit Vipers
Source: Mol Biol Evol. 2022 Jun 20;39(7):msac140. doi: 10.1093/molbev/msac140 (PMC9255381; doi:10.1093/molbev/msac140)
Supplement: msac140_Supplementary_Data [file msac140_supplementary_data.zip › Sup Figure 2.pdf]

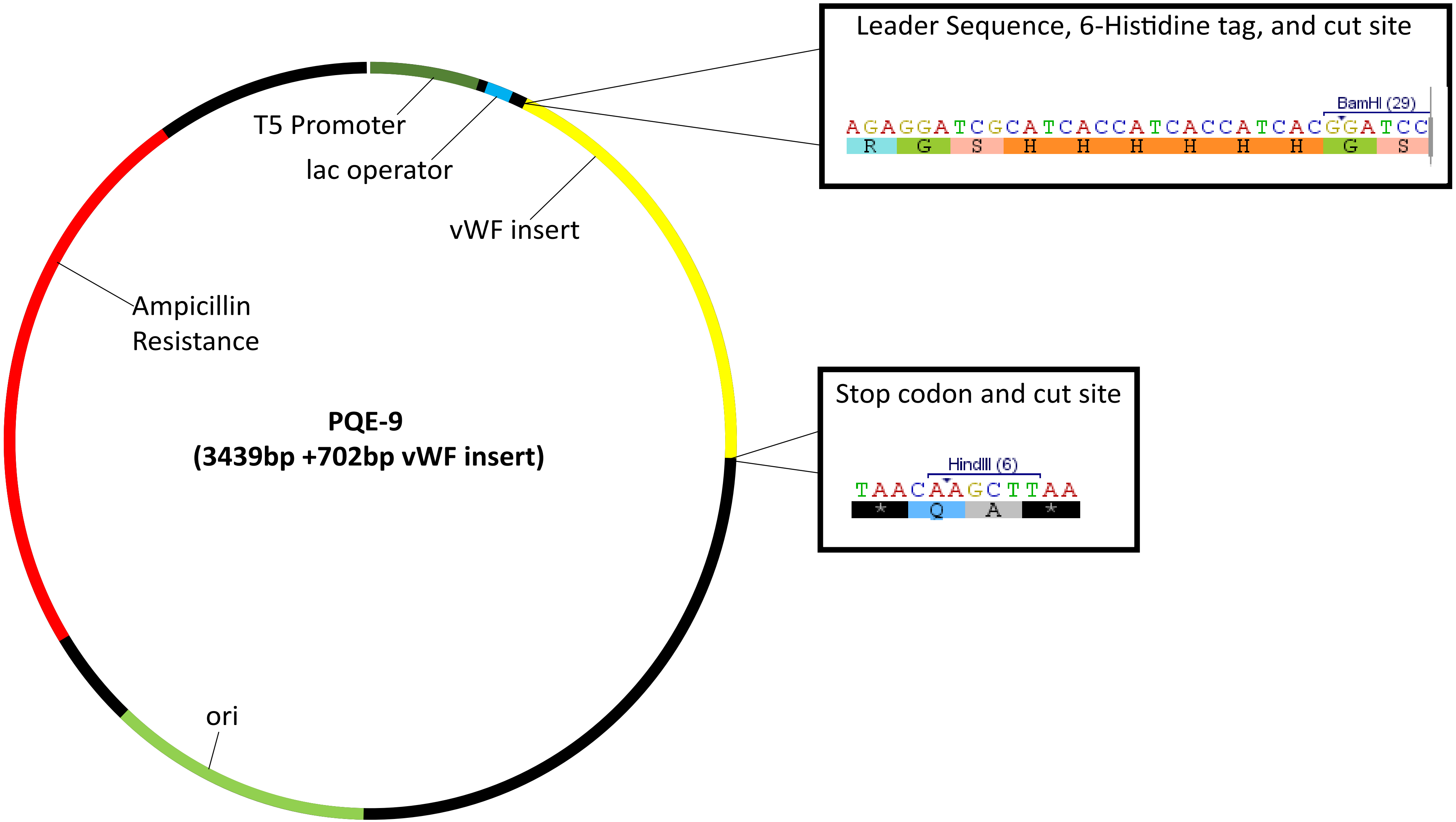

Supplementary Figure 2- Cartoon depiction of the pQE9 vector with the vWF insert pictured. Zoom shows the exact sequences of the leader and terminating sequence. Not all features shown.
