# Supplementary material for: Ancestrally Reconstructed von Willebrand Factor Reveals Evidence for Trench Warfare Coevolution between Opossums and Pit Vipers
Source: Mol Biol Evol. 2022 Jun 20;39(7):msac140. doi: 10.1093/molbev/msac140 (PMC9255381; doi:10.1093/molbev/msac140)
Supplement: msac140_Supplementary_Data [file msac140_supplementary_data.zip › Supp_Fig3.pdf.pdf]

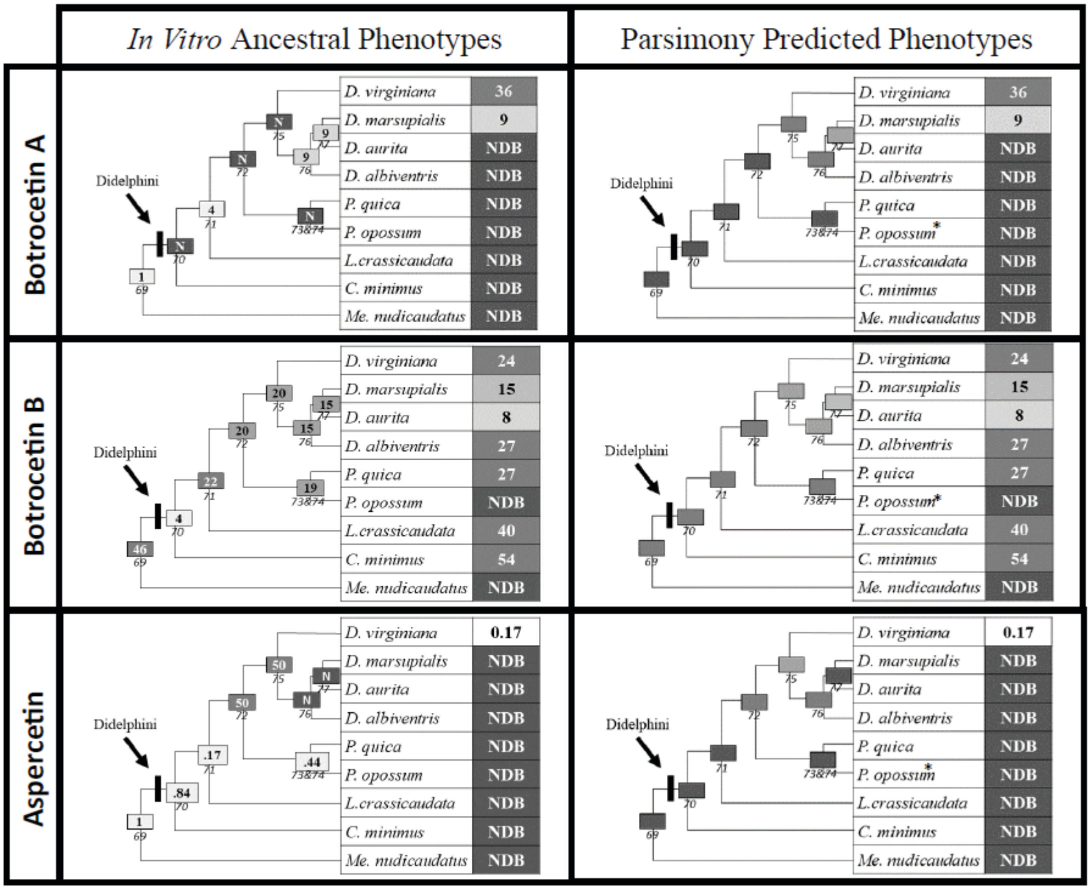

Supplementary Figure 3- Relative binding of ancestral nodes of vWF A1 mapped onto a species topology. On the left is the actual binding capacity of each node measured via biophysical assays of heterologously expressed ancestral protein. Lower values represent stronger binding.   On the right are the predicted phenotypes of the nodes based on a squared change parsimony reconstruction of phenotypes given the binding data from tips. Asterisks indicated identical protein sequences Philander opossum and Philander mcilhennyi collapsed as one tip taxa, subsequent nodes (node 73 and 74) are also identical and collapsed into one node. Heat map indicates loss of binding with darkening shades, with increments of 0-5x, 6-10x, 11-15x, 16-20x, >20x, and NDB. Numbers at each node indicates actual multiple of binding loss relative to human (KD divided by human KD). NDB at tips and N at nodes indicates no detectable binding. Extant relative binding is shown at the tips for reference. Node numbers correspond to Supplementary Figure 1 and are labeled under each node's relative binding.
