# Supplementary material for: Ancestrally Reconstructed von Willebrand Factor Reveals Evidence for Trench Warfare Coevolution between Opossums and Pit Vipers
Source: Mol Biol Evol. 2022 Jun 20;39(7):msac140. doi: 10.1093/molbev/msac140 (PMC9255381; doi:10.1093/molbev/msac140)
Supplement: msac140_Supplementary_Data [file msac140_supplementary_data.zip › Supplementary Methods.pdf]

Supplementary Methods

vWF Polymerase Chain Reaction Protocol:

PCRs were carried out in a 25 µl reaction using 0.5 µl of 10 mM dNTPs, 15 µl ddH$_2$0, 5 µl 5X Phire reaction buffer (Thermo-Scientific), 1 µl each of 10 mM primer solution of forward and reverse primers, 0.5 µl of Phire polymerase (Thermo-Scientific), and 2 µl of template DNA. Primers DvWF_F1 and DvWF_R1 were used for the partial upstream sequence, DvWF_F1 and DvWF_R6 was used for sequencing the complete vWF region (covering residues 475-543) (Supplementary Table 2). Reactions included a 30 second 98° C activation step, after which 35 cycles were programmed as follows: 98° C for 5 seconds, 65° C for 5 seconds, and 72° C for 15 seconds. Amplified PCR product was sequenced at the University of Minnesota Genomics Center for all species. Resulting sequences were edited and assembled in Geneious version 7.1.8. Upstream sequences were accessioned to GenBank as amendments to the original sequence accession, and new sequences were accessioned separately (Supplementary Table 1).

Plasmid Isolation protocol:

To isolate mutant plasmids, ligation product was transformed into chemically competent M15 cells and allowed to recover in LB media at 37° C for 1 hour before being plated on LB-ampicillin plates (50 µg/mL) and grown at 37° C overnight. Single colonies were picked, grown overnight in liquid LB with 100 µg/ml ampicillin, and plasmids were isolated via a QIAprep Spin Miniprep Kit (QIAGEN, Hilden, Germany). Isolated plasmids were checked for correct insert sequence by PCR amplification using 1 µl each of a 10mM primer solution of PQE9 Insert F1 and PQE9 insert R3 0.5 µl 10mM dNTPs, 15 µl ddH$_2$0, 0.5 µl Phire polymerase, and 5 µl 5X Phire buffer (Thermo-Scientific) (Supplementary Table 2). Reactions were run on a thermocycler using a 30 second 98° C activation step, and 35 cycles as follows: 98° C for 5 seconds, 65° C for 5 seconds, and 72° C for 15 seconds. PCR products were Sanger sequenced at the University of Minnesota Genomics Center with the same primers. Sequences were edited and assembled in Geneious version 7.1.8; multi-species sequence alignments were created using

MUSCLE (Edgar 2004). Vectors containing the correct inserted sequences were stored at -20° C for use in overexpression. A complete list of vWF constructs for extant and ancestral species can be found in Supplementary Material (Constructs.phy).

BLItz protocol:

Because curves from multiple concentrations of an analyte are required for accurate equilibrium estimation of binding affinity, each assay used 4-7 concentrations of analyte (venom-CTL), resulting in 4-7 binding curves for each vWF-venom pair (Shah and Duncan 2014). Where possible, replicate assays were run using the same sample or samples made in separate batches. Samples of venom and vWF were stored in separate boxes before and after data collection, and Human vWF A1 was used to spot-check venom protein activity periodically throughout data collection. A complete list of data collected for each vWF and venom protein pair by date can be found in Supplementary Table 3.

Both vWF and venom proteins were diluted into a buffer containing 25 mM Tris, 150 mM NaCl 1% BSA, 0.05% Tween 20, pH 7.4. vWF was diluted into test buffer to an approximate concentration of 200 nM for loading on to the chip. Venom proteins were serially diluted into concentration series which were appropriate for the binding affinity of each ligand-analyte pair.To standardize for K_D differences between antibody and Nickel chips, K_D for human samples (tight binders) were compared between chips and the derived ratio was used to scale binding data gathered from different chips. Thus, Botrocetin B NiNTA results (K_D, k_a, and k_d) were divided by 7.59, bitiscetin HS1K results (K_D, k_a, and k_d) were divided by 7.78.

Per manufacturer recommendations, biosensors were incubated in buffer for ten minutes prior to each test. Kinetic data collection was performed with a 30 second buffer baseline, followed by a 120 second loading step in which 4 µl of vWF A1 was exposed to the biosensor chip surface. This was followed by a second baseline for 30 seconds, an association phase of 60-120 seconds in which 4 µl of various concentrations of venom protein were allowed to associate to the chip, and a disassociation phase

of the same length (either 60 or 120 seconds) where the biosensor was returned to buffer. A sample containing no venom protein was used to calibrate machine noise and subtracted from all curves, as is standard (Sultana and Lee 2015). Association and disassociation times were chosen as sufficient time for the CTL to reach equilibrium association to vWF, as well as disassociation of more than 50% (Sultana and Lee 2015). Nonspecific binding assays were performed by using a blank (buffer with no vWF protein) for loading, and a sample containing the venom protein at up to the second highest concentration used for each curve series on each chip. Nonspecific binding was visually observed as a flat line for each CTL on each chip type, confirming no nonspecific binding, and not further analyzed (Sultana and Lee 2015).

BLItz Pro software optimizes the observed association constant (k_{on}), dissociation constant (k_{off}), and R_{max} (the maximum binding of the venom protein, given the amount of vWF on the biosensor surface) over multiple curves (serial dilutions). This software uses optimized values of k_{on} and k_{off} to calculate an equilibrium constant K_D, where K_D for a given Analyte (A) and Ligand (L) is $\frac{[A][L]}{[AL]} = \frac{koff}{kon}$ = $K_{D,}$ given a 1:1 binding model. A 1:1 binding model was used as both botrocetin and bitiscetin have been shown to bind in a 1:1 conformation with vWF A1 (Maita et al. 2003, Fukuda et al. 2005). BLItz Pro software 1:1 binding model (ForteBio, Pall Corperation). As provided by the manufacturer:

Curve Fitting

The BLItz Pro software version 1.2.1.3 subtracts a zero concentration curve from all curve data. Using a full-fitting model it optimizes for R_{max}, K_a, and K_d given each analyte (venom) concentration using the equations (ForteBio knowledgebase, Pall Corporation):

Association Phase:

$$Y = R_{max}\frac{1}{1 + \frac{k_d}{k_a*[Analyte]}}(1 - e^{-\left(k_a*[Analyte] + k_d\right)t})$$

Disassociation Phase:

$$Y = Y_A e^{-k_d\left(t - t_A\right)}$$

$$Y_A = R_{max}\frac{1}{1+\frac{k_d}{k_a*[Analyte]}}(1-e^{-\left(k_a*[Analyte]+k_d\right)t_A})$$

- Y is the BLI signal in nm, which indicates the level of binding as a nm shift.
- t is time in seconds.
- k_a is the association rate constant.
- k_d is the dissociation rate constant.
- [Analyte] refers to the provided concentration of the analyte in solution.
- R_{max} represents the fitted maximum achievable binding for an analyte to a given level of immobilized ligand on the biosensor surface.
- t_A represents the time at the end of association, which is also the time at the beginning of dissociation.
- Y_A represents the calculated nm shift at the end of association (when time is at t_A)

This full fitted model is used to optimize observed values for R_{max}, K_a, and k_d, which are directly observed from each analyte concentration curve.

Calculation of Binding Affinity

BLItz software subsequently uses these optimized values and the equations below calculate k_{obs}, R_{eq} and K_D for each analyte concentration.

$$k_{obs} = k_a*[Analyte] + k_d$$

$$R_{eq} = R_{max}\frac{k_a*[Analyte]}{k_a*[Analyte]+k_d} = R_{max}\frac{[Analyte]}{[Analyte]+K_D}$$

- k_a is the association rate constant.
- k_d is the dissociation rate constant.
- k_{obs} is the observed rate constant reflecting the overall rate of the combined association and dissociation of the two binding partners.
- [Analyte] refers to the provided concentration of the analyte in solution.
- R_{eq} (R equilibrium) is the fitted binding response value (nm shift) when the binding interaction reaches equilibrium between association and dissociation for a given analyte concentration.
- R_{max} represents the calculated maximum achievable binding for an analyte to a given level of immobilized ligand on the biosensor surface.

References

Edgar, R.C. 2004. MUSCLE: multiple sequence alignment with high accuracy and high throughput. Nucleic Acids Research 32:1792-1797.

Shah, N.B., and T.M. Duncan. 2014. Bio-layer interferometry for measuring kinetics of protein-protein interactions and allosteric ligand effects. J. Vis. Exp. 84: e51383

Sultana A., and J.E. Lee. 2015. Measuring Protein-Protein and Protein-Nucleic Acid Interactions by Biolayer Interferometry. Current Protocols in Protein Science 79(1): 19.25.1-19.25.26
