# Supplementary material for: Ancestrally Reconstructed von Willebrand Factor Reveals Evidence for Trench Warfare Coevolution between Opossums and Pit Vipers
Source: Mol Biol Evol. 2022 Jun 20;39(7):msac140. doi: 10.1093/molbev/msac140 (PMC9255381; doi:10.1093/molbev/msac140)
Supplement: msac140_Supplementary_Data [file msac140_supplementary_data.zip › Supplementary Table 1.pdf]

**Species**	**NCBI Accession #**
Chironectes minimus	FJ159330.1*
Didelphis virginiana	FJ159335.1*
Lutreolina crassicaudata	FJ159342.1*
Philander quica	FJ159362.1*
Philander mcilhennyi	**MN18654**
Philander opossum	FJ159364.1*
Metachirus nudicaudata	FJ159353.1*
Didelphis marsupialis	**MN18653**
Homo sapiens	AAB39987.1
Monodelphis domestica	NW_001582018.1
Monodelphis emiliae	FJ159358.1*
Didelphis aurita	**MN18655-60**
Didelphis albiventris	FJ159333.1*

Supplementary Table 1- List of specimens used for vWF constructs. GenBank accession numbers with asterisks were amended with the newly sequenced upstream portion of vWF for this work. Accession numbers in bold were novel and accessioned uniquely. Sequences not in bold were used directly from GenBank and not re-sequenced.
