# Supplementary material for: Ancestrally Reconstructed von Willebrand Factor Reveals Evidence for Trench Warfare Coevolution between Opossums and Pit Vipers
Source: Mol Biol Evol. 2022 Jun 20;39(7):msac140. doi: 10.1093/molbev/msac140 (PMC9255381; doi:10.1093/molbev/msac140)
Supplement: msac140_Supplementary_Data [file msac140_supplementary_data.zip › Supplementary table 2.pdf]

| Primer name | Primer Sequence |
| --- | --- |
| DvWF_F1 | 5’- TCACTGTGATGGTGTGAACTT-3’ |
| DvWF_R6 | 5’- GTCTGAGCCTTCTAGCACAAA-3’ |
| DvWF_R1 | 5'-ACATTGAACTGAAAGATCGGAAGC-3' |
| PQE9 Insert F1 | 5’- CACAGAATTCATTAAAGAGGAGA-3’ |
| PQE9 insert R3 | 5’-CTGAGGTCATTACTGGATCTATCAACAGGA-3’ |

**Supplementary Table 2**- List of primers used.
