# Supplementary material for: Ancestrally Reconstructed von Willebrand Factor Reveals Evidence for Trench Warfare Coevolution between Opossums and Pit Vipers
Source: Mol Biol Evol. 2022 Jun 20;39(7):msac140. doi: 10.1093/molbev/msac140 (PMC9255381; doi:10.1093/molbev/msac140)
Supplement: msac140_Supplementary_Data [file msac140_supplementary_data.zip › Supplementary Table 3.pdf]

date	molecule	chip	species	K_D [M]	k_a	k_d	Rmax	R equilibrium	R^2
9/14/2016	bitiscetin	NiNTA	Human	1.97E-08	2.07E+04	4.07E-04	5.32E-01	5.28E-01	0.9882
9/14/2016	bitiscetin	NiNTA	*Didelphis virginiana*	1.81E-07	2.95E+04	5.35E-03	3.69E-01	3.22E-01	0.9776
11/1/2016	bitiscetin	NiNTA	Human	1.03E-08	1.02E+05	1.05E-03	4.22E-01	4.16E-01	0.9841
11/1/2016	bitiscetin	NiNTA	Human	1.77E-08	9.83E+04	1.74E-03	2.69E-01	2.57E-01	0.9835
11/2/2016	bitiscetin	NiNTA	*Didelphis virginiana*	8.47E-08	7.06E+04	5.98E-03	5.44E-01	4.88E-01	0.9832
11/3/2016	bitiscetin	NiNTA	*Didelphis virginiana*	9.52E-08	4.63E+04	4.41E-03	4.30E-01	3.81E-01	0.9863
11/12/2016	bitiscetin	NiNTA	Human	1.39E-08	8.01E+04	1.12E-03	7.65E-01	7.51E-01	0.9941
11/15/2016	bitiscetin	NiNTA	Human	1.92E-08	8.74E+04	1.67E-03	1.15E+00	1.10E+00	0.9963
11/15/2016	bitiscetin	NiNTA	*Didelphis virginiana*	9.90E-08	1.19E+05	1.18E-02	7.71E-01	6.80E-01	0.9842
12/9/2016	botrocetin A	HS1K	*Didelphis virginiana*	3.00E-05	2.11E+03	6.33E-02	1.76E+00	1.73E-01	0.9905
12/13/2016	botrocetin A	HS1K	Human	6.50E-07	7.23E+03	4.70E-03	5.34E-01	4.45E-01	0.9794
1/2/2017	botrocetin A	HS1K	Human	6.47E-07	7.41E+03	4.80E-03	3.31E-01	2.37E-01	0.9838
1/2/2017	botrocetin A	HS1K	*Didelphis virginiana*	2.16E-05	3.61E+03	7.78E-02	1.72E+00	1.21E-01	0.9835
5/15/2017	bitiscetin	HS1K	*Chironectes minimus*	2.03E-06	6.79E+04	1.38E-01	4.30E-01	2.38E-01	0.9745
5/18/2017	botrocetin A	HS1K	Human	7.69E-07	9.48E+03	7.29E-03	4.50E-01	3.64E-01	0.9896
5/18/2017	botrocetin A	HS1K	*Monodelphis domestica*	2.00E-04	na	na	na	na	na
5/24/2017	aspercetin	HS1K	Human	1.92E-06	2.49E+04	4.78E-02	4.14E-01	3.47E-01	0.9944
5/24/2017	aspercetin	HS1K	*Metachirus nudicaudatus*	2.00E-04	na	na	na	na	na
5/25/2017	aspercetin	HS1K	*Lutreolina crassicaudata*	2.00E-04	na	na	na	na	na
5/25/2017	botrocetin A	HS1K	*Metachirus nudicaudatus*	2.00E-04	na	na	na	na	na
5/25/2017	botrocetin A	HS1K	*Lutreolina crassicaudata*	2.00E-04	na	na	na	na	na
5/25/2017	botrocetin A	HS1K	*Philander quica*	2.00E-04	na	na	na	na	na
5/25/2017	botrocetin A	HS1K	*Didelphis albiventris*	2.00E-04	na	na	na	na	na
5/30/2017	bitiscetin	HS1K	*Didelphis marsupialis*	9.36E-07	2.27E+04	2.12E-02	9.00E-01	6.55E-01	0.9591

5/30/2017	bitiscetin	HS1K	*Chironectes minimus*	6.03E-06	1.89E+04	1.14E-01	1.07E+00	3.13E-01	0.9846
5/30/2017	botrocetin A	HS1K	*Chironectes minimus*	2.00E-04	na	na	na	na	na
5/30/2017	botrocetin A	HS1K	*Didelphis marsupialis*	6.03E-06	3.03E+04	1.83E-01	3.71E-01	1.30E-01	0.9509
6/1/2017	aspercetin	HS1K	*Chironectes minimus*	2.00E-04	na	na	na	na	na
6/1/2017	aspercetin	HS1K	*Didelphis virginiana*	3.64E-07	4.42E+03	1.61E-03	3.59E-01	3.46E-01	0.9865
6/1/2017	bitiscetin	NiNTA	Human	3.44E-08	2.76E+04	9.51E-04	6.71E-01	6.62E-01	0.997
6/1/2017	bitiscetin	NiNTA	*Didelphis virginiana*	4.80E-07	1.90E+04	9.10E-03	1.46E+00	1.23E+00	0.9948
6/2/2017	aspercetin	HS1K	Human	1.50E-06	3.08E+04	4.63E-02	4.09E-01	3.56E-01	0.9941
6/2/2017	aspercetin	HS1K	Human	3.12E-06	2.01E+04	6.25E-02	3.73E-01	2.84E-01	0.9901
6/2/2017	aspercetin	HS1K	*Monodelphis domestica*	2.00E-04	na	na	na	na	na
6/2/2017	aspercetin	HS1K	*Didelphis marsupialis*	2.00E-04	na	na	na	na	na
6/2/2017	aspercetin	HS1K	*Didelphis albiventris*	2.00E-04	na	na	na	na	na
6/19/2017	aspercetin	HS1K	*Philander quica*	2.00E-04	na	na	na	na	na
6/19/2017	aspercetin	NiNTA	*Philander opossum*	2.00E-04	na	na	na	na	na
6/19/2017	botrocetin A	NiNTA	*Philander opossum*	2.00E-04	na	na	na	na	na
6/27/2017	bitiscetin	NiNTA	*Didelphis albiventris*	9.77E-07	9.09E+03	8.89E-03	3.17E+00	2.28E+00	0.9947
6/27/2017	bitiscetin	NiNTA	*Didelphis marsupialis*	7.91E-07	7.82E+03	6.18E-03	3.99E+00	3.03E+00	0.9973
6/27/2017	bitiscetin	NiNTA	*Monodelphis domestica*	5.70E-07	1.85E+04	1.06E-02	1.27E+00	1.03E+00	0.9928
6/27/2017	bitiscetin	NiNTA	*Metachirus nudicaudatus*	3.10E-06	3.80E+03	1.18E-02	3.56E-01	1.59E-01	0.9141
2/23/2018	botrocetin A	HS1K	Human	7.99E-07	8.51E+03	6.81E-03	4.04E-01	3.25E-01	0.9608
2/23/2018	botrocetin A	HS1K	Node 69	9.79E-07	1.13E+04	1.11E-02	5.53E-01	4.25E-01	0.9721
2/23/2018	botrocetin A	HS1K	*Monodelphis emiliae*	2.00E-04	na	na	na	na	na
2/24/2018	bitiscetin	NiNTA	*Monodelphis emiliae*	1.96E-06	9.50E+03	1.87E-02	2.31E+00	1.29E+00	0.9977
2/24/2018	bitiscetin	NiNTA	Node 69	3.03E-07	1.80E+04	5.45E-03	1.16E+00	1.04E+00	0.9934
2/25/2018	aspercetin	HS1K	Node 69	2.67E-06	1.97E+04	5.26E-02	3.21E-01	2.54E-01	0.9769
3/27/2018	bitiscetin	NiNTA	*Lutreolina crassicaudata*	2.11E-07	1.64E+04	3.47E-03	6.91E-01	6.37E-01	0.9921

3/27/2018	bitiscetin	NiNTA	*Lutreolina crassicaudata*	3.74E-07	1.31E+04	4.89E-03	8.68E-01	7.55E-01	0.9938
3/28/2018	bitiscetin	NiNTA	*Philander quica*	6.95E-07	1.49E+04	1.04E-02	5.88E-01	4.60E-01	0.9925
3/28/2018	bitiscetin	NiNTA	*Philander quica*	1.33E-06	1.05E+04	1.39E-02	5.05E-01	3.30E-01	0.9768
3/28/2018	bitiscetin	NiNTA	*Philander opossum*	2.00E-04	na	na	na	na	na
3/29/2018	botrocetin A	HS1K	*Philander quica*	2.00E-04	na	na	na	na	na
3/29/2018	botrocetin A	HS1K	*Didelphis marsupialis*	6.48E-06	7.31E+03	4.74E-02	7.37E-01	2.46E-01	0.9807
7/16/2018	botrocetin B	HS1K	Human	5.09E-08	5.20E+05	2.65E-02	2.39E-01	2.38E-01	0.9699
12/13/2018	botrocetin A	HS1K	Node 70	2.00E-04	na	na	na	na	na
12/13/2018	botrocetin A	HS1K	Node 71	2.87E-06	3.05E+04	8.75E-02	3.85E-01	2.05E-01	0.9788
12/13/2018	botrocetin A	HS1K	Node 72	2.00E-04	na	na	na	na	na
12/13/2018	botrocetin A	HS1K	Node 73	2.00E-04	na	na	na	na	na
12/16/2018	botrocetin B	HS1K	*Didelphis marsupialis*	7.49E-07	2.22E+05	1.66E-01	1.56E-01	1.13E-01	0.9569
12/16/2018	botrocetin B	HS1K	*Lutreolina crassicaudata*	1.39E-06	9.69E+04	1.35E-01	2.42E-01	2.23E-01	0.9645
12/16/2018	botrocetin B	HS1K	*Lutreolina crassicaudata*	3.00E-06	6.24E+04	1.87E-01	1.30E+02	3.26E+01	0.9394
12/17/2018	botrocetin B	NiNTA	*Chironectes minimus*	2.78E-05	3.14E+03	8.73E-02	5.38E-01	1.97E-01	0.9377
12/17/2018	botrocetin B	HS1K	*Didelphis virginiana*	1.66E-06	1.51E+05	2.51E-01	4.67E-01	4.23E-01	0.9841
12/18/2018	botrocetin B	HS1K	*Didelphis albiventris*	2.63E-06	1.39E+05	3.64E-01	4.61E-01	3.47E-01	0.9784
12/18/2018	botrocetin B	NiNTA	*Didelphis albiventris*	1.06E-05	4.24E+04	4.50E-01	9.04E-01	7.78E-02	0.9917
1/8/2019	botrocetin B	NiNTA	Human	4.99E-07	3.85E+04	1.92E-02	1.39E+00	1.31E+00	0.9812
1/8/2019	botrocetin B	NiNTA	Human	3.72E-07	7.28E+04	2.71E-02	6.10E-01	5.97E-01	0.9892
1/9/2019	botrocetin B	NiNTA	*Didelphis virginiana*	1.33E-05	2.19E+04	2.90E-01	4.71E-01	6.18E-02	0.9626
1/9/2019	botrocetin B	NiNTA	Human	4.18E-07	7.11E+04	2.97E-02	7.67E-01	7.29E-01	0.9895
1/10/2019	botrocetin B	NiNTA	*Didelphis albiventris*	6.91E-06	4.05E+04	2.79E-01	2.26E+00	1.58E+00	0.9928
1/10/2019	botrocetin B	NiNTA	*Didelphis albiventris*	8.16E-06	3.71E+04	3.03E-01	2.64E+00	1.75E+00	0.9916
1/10/2019	botrocetin B	NiNTA	*Didelphis marsupialis*	6.16E-06	2.73E+04	1.68E-01	3.11E+00	2.25E+00	0.9921
1/10/2019	botrocetin B	NiNTA	*Didelphis marsupialis*	6.96E-06	3.85E+04	2.68E-01	3.07E+00	2.14E+00	0.9797

1/10/2019	botrocetin B	NiNTA	*Chironectes minimus*	1.76E-05	1.89E+04	3.32E-01	2.12E-01	1.01E-01	0.9416
1/10/2019	botrocetin B	NiNTA	*Didelphis virginiana*	6.19E-06	3.28E+04	2.03E-01	1.31E+00	9.45E-01	0.9974
1/10/2019	botrocetin B	NiNTA	*Didelphis virginiana*	7.65E-06	3.52E+04	2.70E-01	1.58E+00	1.07E+00	0.9965
1/10/2019	botrocetin B	NiNTA	*Philander opossum*	2.00E-04	na	na	na	na	na
1/15/2019	botrocetin B	NiNTA	*Didelphis aurita*	3.87E-06	5.71E+04	2.21E-01	2.37E-01	1.91E-01	0.9829
1/15/2019	botrocetin B	NiNTA	*Didelphis aurita*	2.64E-06	5.10E+04	1.35E-01	2.63E-01	2.25E-01	0.9843
1/15/2019	botrocetin B	NiNTA	*Metachirus nudicaudatus*	2.00E-04	na	na	na	na	na
1/15/2019	botrocetin B	NiNTA	*Metachirus nudicaudatus*	2.00E-04	na	na	na	na	na
1/15/2019	botrocetin B	NiNTA	*Monodelphis emiliae*	4.92E-05	1.27E+04	6.23E-01	2.09E+00	5.13E-01	0.9954
1/15/2019	botrocetin B	NiNTA	*Monodelphis emiliae*	1.30E-05	3.47E+04	4.51E-01	8.88E-01	4.90E-01	0.9938
1/18/2019	aspercetin	HS1K	Node 72	2.00E-04	na	na	na	na	na
1/18/2019	botrocetin B	NiNTA	Node 69	6.77E-06	2.83E+04	1.91E-01	1.03E+00	7.21E-01	0.9924
1/18/2019	botrocetin B	NiNTA	Node 71	9.14E-06	2.29E+04	2.09E-01	2.79E+00	1.78E+00	0.9978
1/18/2019	botrocetin B	NiNTA	Node 71	9.52E-06	2.35E+04	2.24E-01	3.41E+00	1.56E+00	0.9937
1/18/2019	botrocetin B	NiNTA	Node 72	8.45E-06	2.14E+04	1.81E-01	4.06E+00	2.66E+00	0.9947
1/18/2019	botrocetin B	NiNTA	Node 69	1.25E-05	2.16E+04	2.69E-01	1.77E+00	9.92E-01	0.9965
1/21/2019	botrocetin B	NiNTA	Node 69	3.62E-05	7.76E+03	2.81E-01	2.24E+00	6.86E-01	0.9773
3/3/2019	botrocetin B	NiNTA	*Monodelphis domestica**	1.55E-05	2.32E+04	3.60E-01	1.07E+00	5.46E-01	0.9905
3/3/2019	botrocetin B	NiNTA	*Monodelphis domestica**	1.36E-05	2.73E+04	3.72E-01	7.30E-01	3.94E-01	0.9968
3/3/2019	botrocetin B	NiNTA	Node 69	2.19E-05	1.17E+04	2.57E-01	1.82E+00	7.66E-01	0.9955
3/3/2019	botrocetin B	NiNTA	Node 70	8.39E-07	5.59E+04	4.69E-02	1.14E-01	1.03E-01	0.9823
3/3/2019	botrocetin B	NiNTA	Node 70	2.41E-06	4.71E+04	1.14E-01	1.55E-01	1.35E-01	0.9418
3/3/2019	botrocetin B	NiNTA	*Philander quica**	1.39E-05	2.52E+04	3.51E-01	1.62E+00	5.91E-01	0.9974
3/3/2019	botrocetin B	NiNTA	*Philander quica**	8.65E-06	3.58E+04	3.10E-01	1.16E+00	5.57E-01	0.9966
3/4/2019	aspercetin	HS1K	*Monodelphis emiliae*	2.69E-07	1.03E+05	2.76E-02	2.64E-01	2.08E-01	0.9758
3/4/2019	aspercetin	HS1K	Node 71	3.71E-07	1.77E+04	6.58E-03	1.35E-01	1.30E-01	0.9616

3/4/2019	aspercetin	HS1K	Node 73	9.59E-07	1.40E+04	1.34E-02	1.68E-01	1.54E-01	0.9595
3/4/2019	aspercetin	HS1K	Node 70	1.83E-06	4.47E+04	8.17E-02	9.06E-02	7.66E-02	0.9719
3/4/2019	aspercetin	HS1K	Node 72	1.77E-05	1.73E+04	3.06E-01	3.83E-01	1.38E-01	0.9418
3/4/2019	aspercetin	HS1K	*Didelphis aurita*	2.00E-04	na	na	na	na	na
3/4/2019	botrocetin B	NiNTA	Node 73	5.71E-06	2.65E+04	1.52E-01	2.61E+00	1.92E+00	0.9949
3/4/2019	botrocetin B	NiNTA	Node 73	1.05E-05	1.85E+04	1.95E-01	5.33E+00	4.63E-01	0.9973
3/20/2019	bitiscetin	NiNTA	*Didelphis aurita*	2.15E-07	3.34E+04	7.20E-03	4.08E-01	3.48E-01	0.9777
3/20/2019	bitiscetin	NiNTA	*Didelphis aurita*	6.40E-07	1.77E+04	1.13E-02	5.62E-01	4.47E-01	0.9842
3/21/2019	botrocetin A	HS1K	Human	9.65E-07	7.10E+03	6.85E-03	4.61E-01	3.56E-01	0.98250
3/22/2019	botrocetin A	HS1K	*Didelphis aurita*	2.00E-04	na	na	na	na	na

Supplementary Table 3- All data collection for curve sets by date from oldest to newest. Table is color coded by venom protein. All data is from a globally fit 1:1 binding model performed in BLItz Pro software. Date is the date the assay was performed. Chip types are Nickel coated (NiNTA) or pentahistidine tagged (HS1K). Molecular indicates the venom protein used. Species indicates the species (or node) of vWF used. K_D is given here in [M] units. Off rates (k_a) is 1/Ms, and off rates (k_d) are in 1/s (seconds). Rmax is the maximum response determined from the fit of the binding data (nm), R equilibrium is the calculated response (nm) at equilibrium resulting from globally fit binding data. R^2 is a measure of goodness of fit of globally fit binding data. K_D, k_a, and k_d between chips are scaled --for botrocetin B NiNTA results are divided by 7.59, bitiscetin HS1K results are divided by 7.79. Separately expressed bactches of *M. domestica* and *P. quica* are indicated with an asterisk.
