# Supplementary material for: Ancestrally Reconstructed von Willebrand Factor Reveals Evidence for Trench Warfare Coevolution between Opossums and Pit Vipers
Source: Mol Biol Evol. 2022 Jun 20;39(7):msac140. doi: 10.1093/molbev/msac140 (PMC9255381; doi:10.1093/molbev/msac140)
Supplement: msac140_Supplementary_Data [file msac140_supplementary_data.zip › Supplementary Table 5.pdf]

	Parallel Changes	Divergent Changes	Total
Caluromys philander	635		2
Caluromys lanatus	628	635	2
Gilronia venusta	628	635, 639	3
Metachirus nudicaudatus		635	1
Gracilinanus agilis		635	1
Gracilinanus microtarsus		635	1
Gracilinanus aceramarcae		635	1
Gracilinanus emliliae		635	1
Thylamys venustus	635	628	2
Thylamys pallidior	635		1
Thylamys macrurus	635	628	2
Thylamys pusillus	635	628	2
Marmosops incanus		635	1
Marmosops noctivagus		635	1
Marmosops spGalves		635, 639	2
Marmosops pinheiroi		635	1
Marmosops parvidens		635	1
Tlacuatzin canescens		635	1
Monodelphis scalops		635	1
Monodelphis emiliae	635, 636, 668	631, 633	5
Monodelphis pervuviana	635		1
Monodelphis brevicaudata		628, 630, 631, 635	4
Monodelphis domestica		628, 630, 631, 635	4
Marmosa rubra	628, 630, 635, 636, 668	633, 639	8
Marmosa robinsoni		630, 631, 635	3
Marmosa mexicana		630, 631, 635	3
Marmosa murina		628, 631, 635	3
Marmosa lepida		635	1
Marmosa rutteri	628	630, 631, 635	4
Marmosa paraguayana		635	1
Marmosa demerarae		635	1

Supplementary Table 5- Species outside of Didelphini that have parallel changes (the same amino acid replacement) and/or divergent changes at the same site associated with >10 fold binding loss for venom proteins seen in Table 1. Sites associated with loss of binding by venom protein are: botrocetin A [628, 630, 631, 633, 636, 668], botrocetin B [635], aspercetin [630, 631, 639]. One site highlighted in grey (635) indicates that every species had a change from the known binding state (D-Aspartic Acid) at this site. Rows of species that have associated vWF A1 affinity data are highlighted in grey.
