# Supplementary material for: Ancestrally Reconstructed von Willebrand Factor Reveals Evidence for Trench Warfare Coevolution between Opossums and Pit Vipers
Source: Mol Biol Evol. 2022 Jun 20;39(7):msac140. doi: 10.1093/molbev/msac140 (PMC9255381; doi:10.1093/molbev/msac140)
Supplement: msac140_Supplementary_Data [file msac140_supplementary_data.zip › Supplemetary Table 4.pdf]

Supplementary Table 4- Binding kinetics results for vWF from each species tested against four venom CTLs (two isoforms of Botrocetin, Aspercetin, and Bitiscetin). Values shown are average K_D values (in nM) with standard errors when multiple tests were available. Dashes indicate that no binding curve was recovered between vWF from that species and the referenced venom CTL.

	Botrocetin A	Botrocetin B	Aspercetin	Bitiscetin
Didelphis virginiana	25800±4200	1310±233	364	188±75
Didelphis albiventris	—	1502±389	—	977
Didelphis marsupialis	6260 ±225	825±49	—	791
Didelphis aurita	—	429±81	—	427±212
Philander quica	—	67656±66199	—	1010±318
*Philader opossum**	—	—	—	—
Lutreolina crassicaudata	—	2200±805	—	293±82
Chironectes minimus	—	2990±671	—	518±257
Metachirus nudicaudatus	—	—	—	3100
Monodelphis domestica	—	1916±125	—	570
Monodelphis emiliae	—	4090±2380	269	1960
Homo sapiens	766 ± 5.84	55.15 ± 3.74	2180 ± 485	19.2 ± 0.337

*failure to bind for this species may be attributable to a misfolded synthetic vWF protein.
